# Morphological convergence in a Mexican garter snake associated with the ingestion of a novel prey

**DOI:** 10.1002/ece3.3265

**Published:** 2017-08-02

**Authors:** Javier Manjarrez, Constantino Macías Garcia, Hugh Drummond

**Affiliations:** ^1^ Facultad de Ciencias Universidad Autónoma del Estado de México Toluca State of Mexico Mexico; ^2^ Instituto de Ecología Universidad Nacional Autónoma de México Mexico DF Mexico

**Keywords:** crayfish, dentition, feeding niche, head structure, *Regina*, *Thamnophis*

## Abstract

Morphological convergence is expected when organisms which differ in phenotype experience similar functional demands, which lead to similar associations between resource utilization and performance. To consume prey with hard exoskeletons, snakes require either specialized head morphology, or to deal with them when they are vulnerable, for example, during molting. Such attributes may in turn reduce the efficiency with which they prey on soft‐bodied, slippery animals such as fish. Snakes which consume a range of prey may present intermediate morphology, such as that of Thamnophiine (Natricinae), which may be classified morphometrically across the soft–hard prey dietary boundary. In this study, we compared the dentition and head structure of populations of *Thamnophis melanogaster* that have entered the arthropod–crustacean (crayfish)‐eating niche and those that have not, and tested for convergence between the former and two distantly related crayfish specialists of the genus *Regina* (*R*. *septemvittata* and *R. grahamii*). As a control, we included the congener *T. eques*. Multivariate analysis of jaw length, head length, head width, and number of maxillary teeth yielded three significant canonical variables that together explained 98.8% of the variance in the size‐corrected morphological data. The first canonical variable significantly discriminated between the three species. The results show that head dimensions and number of teeth of the two *Regina* species are more similar to those of crayfish‐eating *T. melanogaster* than to non‐crayfish‐eating snakes or of *T. eques*. It is unclear how particular head proportions or teeth number facilitates capture of crayfish, but our results and the rarity of soft crayfish ingestion by *T. melanogaster* may reflect the novelty of this niche expansion, and are consistent with the hypothesis that some populations of *T. melanogaster* have converged in their head morphology with the two soft crayfish‐eating *Regina* species, although we cannot rule out the possibility of a morphological pre‐adaptation to ingest crayfish.

## 1 INTRODUCTION

Some of the most compelling test cases for adaptive evolution involve morphological convergence (Schluter, 2000), which is predicted to evolve when organisms experience similar functional demands on their phenotype (Schluter, 2000; Vincent, Brandley, Herrel, & Alfaro, 2009). If resource use or other environmental factors impose demands on performance, morphological convergence is predicted to occur (Ruber & Adams, 2001; Winemiller, Kelso‐Winemiller, & Brenkert, 1995).

Snakes are very good subjects for studying feeding morphology because their head is directly involved in feeding (Cundall & Rossman, 1984; Dwyer & Kaiser, 1997). They consume their prey whole; therefore, some morphological attributes of their typical prey should be associated with trophic morphology (Hampton, 2011, 2013; Mori & Vincent, 2008; Vincent et al., 2009). To consume prey with external body features such as the hard exoskeletons of arthropods, snakes require specialized morphology (e.g., piercing teeth) or behavior such as targeting arthropods when they are vulnerable, such as when molting, as the hard exoskeleton is both slippery to grasp and hard to pierce.

**Figure 1:**
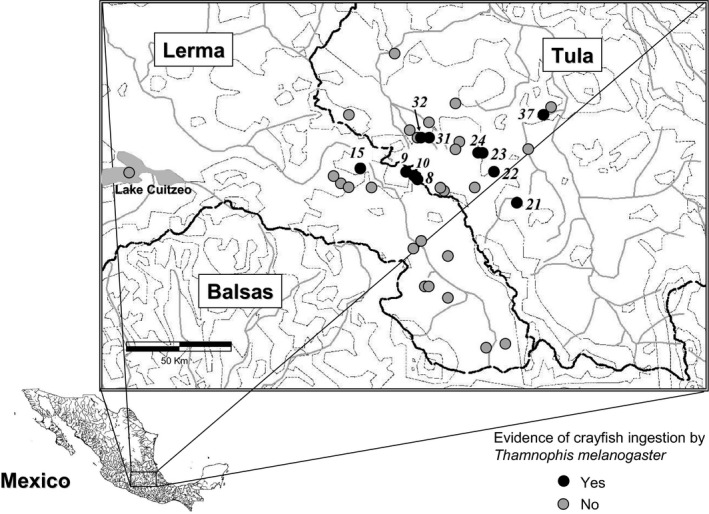
Tula and Lerma drainages where snake *Thamnophis melanogaster* (Natricinae Thamnophiine) consumes crayfish, *Cambarellus montezumae*. Black dashed lines are watershed boundaries; thin dotted lines are 500‐m contour lines, and gray continuous lines are rivers

The tribe Thamnophiine (family Natricinae) comprising North American semi‐aquatic snakes, includes *Thamnophis melanogaster* (Mexican black‐bellied garter snake), an aquatic dietary specialist that is sympatric with a freshwater crustacean, the crayfish *Cambarellus montezumae,* but eats crayfish only in 3.0% of the area of sympatry (Manjarrez, Macías Garcia, & Drummond, 2013; Figure 1). The 35% of prey consumed by *T. melanogaster* were crayfish eaten only when recently molted, so with the exoskeleton as yet unhardened (Manjarrez et al., 2013). Extensive dietary studies of *Thamnophis* species have failed to reveal crayfish ingestion, except in a rare record for *T. proximus* (0.8% of individuals with crayfish in stomachs; Hampton & Ford, 2007). Therefore, the rarity of crayfish ingestion in the focal cluster of populations of *T. melanogaster* (Alfaro & Arnold, 2001; de Queiroz, Lawson, & Lemos‐Espinal, 2002) suggests crayfish eating represents a niche invasion that has not yet expanded to more populations.

Two Thamnophiine species of the genus *Regina* eat newly molted crayfish, which are soft‐bodied (Gibbons & Dorcas, 2004; Godley, 1980; Mushinsky, Hebrard, & Vodopich, 1982). Therefore, we hypothesized that the seemingly recent dietary convergence of some populations of *T. melanogaster* with (allopatric) *Regina* (Hibbitts & Fitzgerald, 2005; McVay & Carstens, 2013) may have led to morphological convergence associated with the demands of finding and capturing hidden soft crayfish.

We explored possible morphological differences in dentition and head structure within *T. melanogaster* by comparing individuals from crayfish‐eating versus non‐crayfish‐eating populations and included in this comparison both soft crayfish‐eating *Regina* species and the aquatic generalist *Thamnophis eques* (Mexican garter snake). *Thamnophis eques* is sympatric with *T. melanogaster* over most of its range (Rossman, Ford, & Siegel, 1996) and represents a control for geographic determinants of head morphology. We predicted a morphological convergence between crayfish‐eating *T. melanogaster* and *Regina* species that specialize in eating soft crayfish.

Consuming soft crayfish may not require specialized teeth, but because of their vulnerability during the molt, crayfish seek refuge and must be sought in burrows and crevices, which would impose different demands on the head morphology of a snake that often preys in the open and guides its strikes visually (Drummond, 1983; Macías Garcia & Drummond, 1995). Dwyer and Kaiser (1997) proposed that Thamnophiine species might be classified morphometrically across the soft–hard prey dietary boundary. They concluded that the soft crayfish‐eating species of *Regina* have skulls of similar dimensions to those of two Thamnophiine species of *Nerodia*, which feed mainly on soft prey (fish; Mushinsky & Hebrard, 1977; Mushinsky & Lotz, 1980), whereas the skulls of hard crayfish eating of *Regina* were different (larger/thicker). It has been proposed that the elongated skull morphology of garter snakes (*Thamnophis*) is associated with the ingestion of soft prey (Britt, Clark, & Bennett, 2009; Savitzky, 1983).

Whereas *R. septemvittata* (Queen snake) and *R. grahamii* (Graham's crayfish snake) eat newly molted crayfish, congeners *R*. *alleni* and *R. rigida* primarily eat hard, nonmolted crayfish (Franz, 1977). Both eaters of newly molted crayfish have shorter and narrower heads than their hard crayfish‐eating congeners (Dwyer & Kaiser, 1997; Nakamura & Smith, 1960), and their teeth are sharp, curved, and oriented backwards as in most generalist relatives Thamnophiine (Myer, 1987; Nakamura & Smith, 1960), contrasting with the more rounded back teeth (to hold hard prey) of *R*. *alleni* and *R*. *rigida* (Nakamura & Smith, 1960; Rossman, 1963).

*Thamnophis melanogaster* is a snake that specializes in underwater foraging and feeds mainly on soft‐bodied aquatic prey such as fish (ca. 50%), tadpoles and leeches. It has a narrow head (Rossman et al., 1996), similar to that of other species that feed on aquatic soft prey (Dwyer & Kaiser, 1997; Hibbitts & Fitzgerald, 2005), and curved, pointed, and backward‐directed maxillary teeth, suitable for piercing through soft skin (Rossman et al., 1996). This species is located within the monophyletic group of garter snakes, whereas *Regina* is polyphyletic with respect to other thamnophiines (Alfaro & Arnold, 2001; Guo et al., 2012; McVay & Carstens, 2013; de Queiroz et al., 2002). This suggests that crayfish ingestion has arisen independently among *Regina* species via evolutionary convergence associated with the ingestion of soft‐versus‐hard crayfish. *Thamnophis eques* feeds on soft prey, primarily leeches, fishes, and frogs (Table 1; Macías Garcia & Drummond, 1988; Drummond & Macías Garcia, 1989; Rossman et al., 1996).

**Table 1:** Mean snout–vent length (SVL ± 1 *SD*, range) of the species/morphs *Regina grahamii, Regina septemvittata, Thamnophis eques,* and two dietary morphs of *Thamnophis melanogaster* (Natricinae Thamnophiine) and their reported prey

Species	*n*	Snout–vent length ± *SD*, (range)	Prey	Reference of prey reported in the diet
*Regina grahamii*	19	30.8 ± 19.8 (18.0–77.0)	Newly molted soft crayfish	Burghardt (1968), Mushinsky and Hebrard (1977), Godley, McDiarmid, and Rojas (1984)
*Regina septemvittata*	81	29.6 ± 14.9 (11.5–65.0)	Newly molted soft crayfish	Burghardt (1968); Godley et al. (1984)
*Thamnophis melanogaster*				
Noncrayfish eating	88	39.5 ± 9.7(19.3–59.5)	Leeches, worms, fish, tadpoles	Manjarrez et al. (2013)
Crayfish eating	80	38.4 ± 11.1 (15.0–56.5)	Leeches, worms, fish, tadpoles, crayfish	Manjarrez et al. (2013)
*Thamnophis eques*	42	55.8 ± 11.5 (31.5–79.0)	Leeches, frogs, fish, and salamanders	Macías Garcia and Drummond (1988), Drummond and Macías Garcia (1989), Manjarrez (1998)

*Regina* and *Thamnophis* are two genera of semi‐aquatic North American snakes (Natricinae Thamnophiine). *Thamnophis* snakes were collected at ponds and rivers in two watersheds in Central Mexico, while *Regina* were museum specimens (see Materials and Methods).

## 2 MATERIALS AND METHODS

We measured 80 crayfish‐eating *T. melanogaster* individuals from 10 populations (Manjarrez  al., 2013) and 88 non‐crayfish‐eating individuals from 29 populations adjacent to the crayfish‐eating populations (Table 1). All snakes were captured in the wild. In addition, we examined 19 specimens of *R. grahamii* and 81 of *R*. *septemvittata* at the Florida Museum of Natural History, University of Florida (Table 1). We also included 42 *T. eques* (Table 1). Snakes were mostly adults or of a size close to that of the adults (Table 1; Appendix 1).

Four variables were used to characterize head structure: (1) jaw length (distance from the posterior edge of the posterior‐most supralabial scale to the anterior tip of the rostrum; King, 2002), (2) head length (distance from the snout tip to the posterior‐most portion of the parietal bone), (3) head width (widest part measured while applying pressure on the posterior portion of the head to spread the quadrates and mandibles laterally; Miller & Mushinsky, 1990), and (4) number of maxillary teeth. Although often used in similar studies (King, 2002; Miller & Mushinsky, 1990), we did not use gape index in our analyses because this is a composite of several of the above measures. An exploratory analysis showed that gape index (computed as the area of an ellipse with major and minor axes equal to jaw length and head width; Miller & Mushinsky, 1990) is highly correlated with the three head measures in *T. eques* and in the two feeding morphs of *T. melanogaster* (Appendix 2). Accordingly, this index does not add information to the analysis beyond that provided by jaw length, head length, and head width (King, 2002). We also measured snout–vent length (SVL, Table 1) and recorded the snake gender.

### 2.1 Statistical analyses

We ascertained whether head measurements differed between sexes. As the head variables are influenced by snake size, sexes were compared using one ANCOVA for each species (*n* = 5) and head measurement (*n* = 4), entering SVL as a covariate (*n* = 20 ANCOVAs; see Appendix 3). In general, these comparisons did not indicate differences between sexes (ANCOVA *F* values range from 0.005 to 3.4, with *p* values from .06 to .94), except in only three of the 20 comparisons (Appendix 3). Consequently, in the multivariate tests described below, the two sexes were pooled.

To verify whether tooth number and head shape in crayfish‐eating populations of *T. melanogaster* are similar to those of *R*. *septemvittata* and *R. grahamii*, we conducted a discriminant function analysis with stepwise selection of variables. The initial explanatory variables were the residuals from linear regressions of head length, head width, and jaw length (all log‐transformed because of the lack of homoscedasticity and skewed distributions) and number of teeth, on SVL. The grouping variable was snake species/dietary morph (*R*. *septemvittata*,* R. grahamii, T. eques, T. melanogaste* crayfish‐eating*, T. melanogaste* noncrayfish eating). We compared the canonical variates among groups using one‐way ANOVAs and explored the distribution of the groups' means within the multivariate morphological space. Tests were performed using with Statistica software (ver. 8.0 StatSoft, Tulsa, Oklahoma, USA) and NCSS 10 Statistical Software (2015; NCSS, LLC. Kaysville, Utah, USA).

## 3 RESULTS

The residuals of number of teeth and jaw length, head length, and head width on SVL contributed significantly to the discriminant function (all *p* < .000001), which correctly classified 64.8% of the 310 snakes on the basis of four significant canonical variables which explained 100% of the variance in the data (Table 2). As the means in canonical variable (CV) 1 (59% of variance explained; Table 3) for the two *Regina* species are virtually identical, the overall canonical analysis did not distinguish between them; it classified all individuals as *R*. *septemvittata*, except for one individual of each species which, interestingly, were classified as crayfish‐eating *T. melanogaster*. Sixty‐six percent of *T. eques* were correctly classified, and the rest were assigned indistinctly to *R*. *septemvittata* and to the two feeding morphs of *T. melanogaster* (Table 2). Among non‐crayfish‐eating *T. melanogaster* 53.4% of individuals were correctly classified, 16% were mistakenly classified as *Regina*, and only 4.5% were mistaken for *T. eques*; the equivalent figures for their crayfish‐eating congeners were 59%, 8.8%, and 5%, respectively (Table 2). Only about one‐quarter of *T. melanogaster* individuals were incorrectly classified as belonging to the alternative dietary morph (26% and 27.5% for noncrayfish eating and crayfish eating respectively), compared to only 18% of *Regina* individuals being incorrectly assigned to the wrong species.

**Table 2:** Number of snakes classified as *Regina grahamii, R. septemvittata, Thamnophis eques*, crayfish‐eating and non‐crayfish‐eating *T. melanogaster* (Natricinae Thamnophiine) by a discriminant function analysis performed using the residuals from linear regressions of number of teeth and three log‐transformed head shape variables, on SVL

True species	*n*	Classified as				
		*R. grahamii*	*R. septemvittata*	*T. melanogaster*		*T. eques*
				Crayfish eating	Noncrayfish eating	
*Regina grahamii*	19	0	18	1	0	0
*Regina septemvittata*	81	0	80	1	0	0
*Thamnophis melanogaster*						
Crayfish eating	81	0	7	47	23	4
Noncrayfish eating	88	0	14	23	47	4
*T. eques*	42	0	6	5	4	27

Wild‐caught *Thamnophis* and museum *Regina* specimens were used (see Materials and Methods).

**Table 3:** Canonical coefficients from a discriminant analysis to assort individual snakes belonging to *Regina grahamii, R. septemvittata*,* Thamnophis eques,* and *T. melanogaster* (Natricinae Thamnophiine) from two dietary morphs; crayfish eating and noncrayfish eating (see Table 2)

Morphological variable	CV 1	CV 2	CV 3
Head width	−0.178	−0.260	−0.575
Head length	−0.114	0.259	−0.021
Jaw length	0.296	−0.473	0.136
Number of teeth	−0.195	−0.049	0.159
Eigenvalue	1.005	0.555	0.120
Proportion of variance explained	59.1	32.6	7.1
Cumulative variance explained	59.1	91.7	98.8
One‐way ANOVA *F* (*df*)	29.8 (16, 923)	21.9 (9, 737)	10.5 (4, 608)
*p*	<.001	<.001	<.001

CVs are linear functions of the original morphological variables (jaw length, head length, head width, and number of maxillary teeth), each multiplied by a canonical coefficient. Measures are from wild‐caught *Thamnophis* and museum *Regina* specimens (see Materials and Methods).

**Figure 2:**
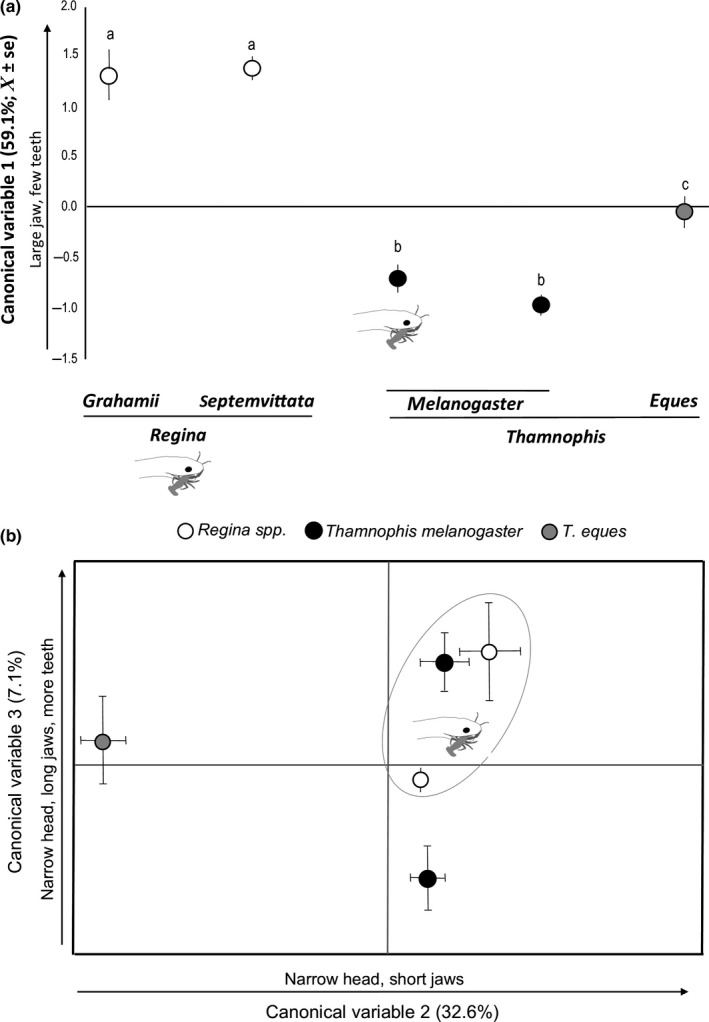
Principal canonical variates obtained from a discriminant function analysis of morphological variation among snake species *Regina septemvittata*,* Regina grahamii, Thamnophis eques,* and *Thamnophis melanogaster* (Natricinae: Thamnophiine) with two dietary morphs, crayfish eating and noncrayfish eating. *Thamnophis* snakes were captured in the wild at two Mexican drainages, while *Regina* were museum specimens (see Materials and Methods). (a) Principal canonical variables (CV)1. Equal letters represent statistical similarity when we compared the canonical variates among groups (one‐way ANOVA). (b) Plotting the Principal canonical variables 2 versus 3 reveal morphological proximity between the crayfish‐eating morph of *Thamnophis melanogaster*, and the two species in genus *Regina* which also prey on newly molted crayfish

Values of CV1 obtained from the discriminant function analysis of morphological variation among means of snake species (which explained 59% of the variance) increased with jaw and head length, and decreased with head width and number of maxillary teeth, according to the coefficients shown in Table 3, and they differed significantly between genera and between *Thamnophis* species, but not between *Regina* species, nor between *T. melanogaster* dietary morphs (Figure 2a; Table 4). Values of CV2, which explained about one‐third (32.6%) of the variance, increased with head length and decreased with jaw length and head width (Table 3). Thus, high values of CV2 depict a slender‐headed snake with a small mouth; hence, it separated (with very large, negative values) stout‐headed *T. eques* from the rest (Table 3). Values in the third canonical variable (CV3), which explained 7% of the variance, decreased with head width while increasing with number of teeth and jaw length (Table 3). On CV3, the two dietary morphs of *T. melanogaster* differed significantly (Table 4, Figure 2b), with crayfish eating also being significantly different from *R*. *septemvittata* and noncrayfish eating also differing significantly from *R. grahamii* (and from *T. eques*; Table 4). Because differences in CV3 (or CV2) are not significant between the two species of *Regina*, it is possible in the plot of the second and third canonical variables to define a morphological space that is shared by both species of *Regina* and by the crayfish‐eating populations of *T. melanogaster* (Figure 2b).

**Table 4:** Canonical means used in the classification of individual *Regina grahamii, R. septemvittata*,* Thamnophis eques*, crayfish‐eating and non‐crayfish‐eating *T. melanogaster* (Natricinae: Thamnophiine) by a discriminant function analysis based on jaw length, head length, head width, and number of maxillary teeth (see Tables 2 and 3)

Snake species/morph	CV 1	CV 2	CV 3
*Regina grahamii*	1.320	0.634	0.458
*Regina septemvittata*	1.399	0.198	−0.060
*Thamnophis melanogaster*			
Crayfish eating	−0.962	0.350	0.414
Noncrayfish eating	−0.679	0.243	−0.458
*Thamnophis eques*	−0.041	−1.847	0.0817

*Thamnophis* (wild‐caught) and *Regina* museum specimens were used (see Materials and Methods).

After correcting for body size, we found no difference in the number of maxillary teeth between the two *Regina* species, but we found difference between the two morphs of *T. melanogaster*. Crayfish‐eating *T. melanogaster* had 2.28 more teeth than non‐crayfish‐eating conspecifics (Student‐*t*_187_ = 2.92, *p *=* *.001), which themselves had 6.0 more teeth than *R. grahamii* and 6.3 more than *R*. *septemvittata* (*F*_1,190_ = 227.6, *p *=* *.0001). We found no difference in the number of maxillary teeth between *T. eques* and either morphs of *T. melanogaster*.

## 4 DISCUSSION

We found a large overlap in head morphology and number of teeth between the several species/morphs examined, yet we also found evidence consistent with the hypothesis that the head morphology of soft crayfish‐eating *T. melanogaster* should more closely resemble that of the two soft crayfish‐eating species of *Regina* than that of non‐crayfish‐eating conspecifics.

Crayfish ingestion in only some locations can be explained by subtle environmental differences between localities (Arnold, 1981), for example, spatiotemporal availability of crayfish or differences in use of microhabitats by *T. melanogaster*. However, a sampling suggests that, if anything, crayfish are more abundant in ponds where snakes do not eat them that in ponds where they do (Appendix 4).

Although significant, the magnitude of the apparent morphological convergence between crayfish‐eating *T. melanogaster* and the two *Regina* species is small. This may be because invasion of this dietary niche is recent, thus even if challenging, crayfish consumption has not had time to shape head and tooth morphology. Alternatively, the selective pressures from soft crayfish predation on head/tooth morphology could be weak, for instance because crayfish‐consuming populations mostly feed on other prey such as fish, tadpoles, and leeches (cf., Forsman & Shine, 1997; Manjarrez et al., 2013). Additionally, other adaptive demands on head morphology may be more important (Rossman & Myer, 1990), while optimal capture and handling of crayfish may require only minor morphological modification (both in *T. melanogaster* and *R*. *septemvittata* and *R*. *grahamii*). Indeed, both *Regina* species have been described as having head and tooth morphologies similar to those of generalist Thamnophiinae snakes (Dwyer & Kaiser, 1997), suggesting that specializing on crayfish does not induce major morphological adaptation.

Snakes preying on soft crayfish may occasionally attack slightly harder ones as these occupy the same refuges and their surface chemicals are capable of eliciting a predatory response (Manjarrez, 2003). If occasionally successful, these attacks could select for morphological adjustments to profit from such encounters. Weak selective pressure of this kind may be operating in both soft crayfish‐eating *Regina* species and in soft crayfish‐eating *T. melanogaster*, slowly yielding minor convergence.

The small effect size of our evidence for convergence may reflect the novelty of this niche expansion by *T. melanogaster* (Arnold, 1981). No phylogeographic analysis has been made, but the restricted geographic expansion of crayfish ingestion (only 3% of the total area of sympatry of crayfish and *T. melanogaster*; Manjarrez et al., 2013) and its location close to the southern limit of the snake's distribution (the Natricinae originated further north) suggests that crayfish ingestion by *T. melanogaster* is a recent development (Lozoya, 1988).

It has been proposed that dental morphology in snakes is associated with dietary preferences (e.g., Britt et al., 2009). *Thamnophis melanogaster* has maxillary teeth that are curved, pointed, and oriented to pierce soft prey such as vulnerable molting crayfish. Only a few snake species ingest hard preys, and they have specialized teeth. For example, *R. alleni* and *R. rigida* have maxillary teeth with rounded tips for handling hard crayfish (Dwyer & Kaiser, 1997), whereas *Storeria* has long maxillary teeth that allow the extraction of land snails from their shells (Rossman & Myer, 1990). The higher number of maxillary teeth in crayfish‐eating *T. melanogaster* (34.1 ± 3.9 teeth) compared with their congeners (32.2 ± 4.9 teeth) and soft crayfish *Regina* is unlikely to be an adaptation to ingest soft crayfish *per se*, as this runs against the trend of fewer maxillary teeth. We should, however, not dismiss too readily the possibility that having more teeth is adaptive when preying on soft crayfish, because different combinations of teeth number, head, and jaw morphology may represent equivalent mechanical solutions to the same problem (see also Arnold, 1993).

The limited scope of morphological microevolution associated with adopting a crayfish diet could also be interpreted as evidence for *T. melanogaster* being morphologically pre‐adapted to ingest crayfish. Our multivariate analysis supports this hypothesis because in relation to CV2, which explained a third of the variance in the original variables, *Regina* species and *T. melanogaster* cluster together and away from *T. eques* (Figure 2). *Thamnophis* is a monophyletic group that originated in the Mexican highlands ~5–6 million years ago (Mao & Dessauer, 1971; de Queiroz et al., 2002), whereas *Regina* is a polyphyletic group first found in North America 4–5 million years ago (Guo et al., 2012), making it more recently evolved than *Thamnophis*. Consequently, crayfish consumption by *T. melanogaster* could represent recent dietary convergence (analogy) with *Regina* rather than homology resulting from the common ancestor of *Regina* and *T. melanogaster* and more primitively shared with *T. eques*. The rarity of soft crayfish ingestion within populations *T. melanogaster* supports the hypothesis of analogous behavior, and it is more likely a phenomenon of invasion of a new feeding niche in an aquatic diurnal species (Hibbitts & Fitzgerald, 2005).

In conclusion, our analyses suggest that *T. melanogaster* shows morphological convergence in head and tooth parameters with two *Regina* species, potentially associated with the ingestion of a novel prey, newly molted crayfish, by the genus *Thamnophis*.

## CONFLICT OF INTEREST

The authors have no conflict of interest on the manuscript.

## Appendix 1 Number (and %) of juvenile and adult snakes of each species/morph included in the analyses. In these viviparous snakes, the criterion to decide whether one individual is adult or not is normally drawn from the size (snout–vent length; SVL, in cm) of the smallest recorded pregnant female

### 1 .1

**Table:**

Species/morph	Juvenile	Adult	Threshold SVL
*Thamnophis melanogaster*			
Crayfish eating	24 (30%)	56 (70%)	33
Noncrayfish eating	21 (24%)	67 (76%)	33
*Thamnophis eques*	3 (7%)	39 (93%)	39
*Regina grahamii*	11 (58%)	8 (42%)	31
*Regina septemvittata*	48 (59%)	33 (41%)	35.5

## Appendix 2 Pearson correlation coefficients between Gape index (Miller & Mushinsky, 1990) and 1) jaw length, 2) head length, and 3) head width of snake species/dietary ecotypes *Regina septemvittata*,* R. grahamii*,* Thamnophis eques*, and crayfish‐eating and non‐crayfish‐eating *T. melanogaster*

### 2 .1

**Table:**

Snake species/morph	*df*	Jaw length	Head length	Head width
*Regina grahamii*	17	0.008	−0.166	0.032
*Regina septemvittata*	79	0.191	0.195	0.105
*Thamnophis melanogaster*				
Crayfish eating	78	0.960*	0.733*	0.866*
Noncrayfish eating	86	0.957*	0.721*	0.821*
*Thamnophis eques*	40	0.984*	0.944*	0.967*

## Appendix 3 ANCOVA *F* (and probability) of within species pairwise slope comparisons between sexes of jaw length, head length, head width (all log‐transformed) and number of maxillary teeth as dependent variables, and SVL as covariate. Only one contrast is significant (in bold) after correcting for multiple (*n* = 4) comparisons per species

### 3 .1

**Table:**

Snake species/morph	*df*	Jaw length	Head length	Head width	Number of maxillary teeth
*Regina grahamii* 8 males: 11 females	1, 16	0.32 (0.58)	0.13 (0.73)	0.10 (0.76)	0.28 (0.87)
*Regina septemvittata* 28 males: 30 females	1, 78	0.90 (0.35)	3.0 (0.06)	0.50 (0.47)	0.92 (0.34)
*Thamnophis melanogaster*					
Crayfish eating 54 males: 40 females	1, 77	0.68 (0.41)	1.50 (0.21)	3.40 (0.07)	0.005 (0.94)
Noncrayfish eating 52 males: 40 females	1, 85	2.30 (0.13)	0.83 (0.36)	7.04 (0.01)	0.38 (0.53)
*Thamnophis eques* 23 males: 19 females	1, 39	4.40 (0.04)	4.70 (0.03)	1.0 (0.33)	0.45 (0.50)

## Appendix 4 Mean abundance of crayfish (*Cambarellus montezumae*) in ponds from of Mexican drainages where *Thamnophis melanogaster* consumes crayfish and where it does not consume such prey. We sampled crayfish sporadically on repeated visits during the rainy season (June to October). We measured crayfish abundance by hauling a seine net (2.8‐m‐long, 5‐mm mesh) toward the pond shore at 10 sites. Abundance is expressed as the average number of crayfishes per haul/location

### 4 .1

**Table:**

*Thamnophis melanogaster*	Mean crayfish abundance in pond ± *SD*	Number of locations sampled	Student's *t* test
Crayfish eating	0.66 ± 1.2	9	*t *=* *0.58 *p** ***=*** ***.23
Noncrayfish eating	2.08 ± 3.03	8	
